# Physiology: Its Place in General Education
*Animal Physics; or the Body and its Functions familiarly explained. By Dionysius Lardner, D.C.L., formerly Professor of Natural Physiology and Astronomy in University College, London. London: Walton and Maberly. 1857.Animal Physiology for Schools. By Dionysius Lardner, D.C.L. With 190 Illustrations. London: Walton and Maberly. 1858.Atlas of Human Anatomy and Physiology. By William Turner, M.R.C.S. Eng., Demonstrator of Anatomy in the University of Edinburgh. Selected and arranged under the superintendence of John Goodsir, F.R.C.S., L. and E. Engraved and printed in colours by W. and A. K. Johnston. Edinburgh: W. and A. K. Johnston. 1857.Handbook to Atlas of Human Anatomy and Physiology. By W. Turner, M.R.C.S.Eng. Edinburgh: W. and A. K. Johnston. 1857.Man; his Structure and Physiology; popularly explained and demonstrated. By R. Knox, M.D., F.R.S.E., Lecturer on Anatomy, etc. With eight movable dissected Coloured Plates, and five Woodcuts. London: H. Baillière. 1857.A Catechism of the Physiology and Philosophy of Body, Sense, and Mind; for use in Schools and Colleges, and in Private Study. By T. Wharton Jones, F.R.S. London: Churchill. 1858.Note on Teaching Physiology in the Higher Schools. By Henry W. Acland, M.D., F.R.S., Regius Professor of Medicine in the University of Oxford. Pamphlet. J. H. and J. Parker. 1858.The Museum of Science and Art. Edited by D. Lardner, D.C.L. Articles—Air, Fire, the Eye, Spectacles, Man, Instinct, and Intelligence.


**Published:** 1858-04

**Authors:** 


### PHYSIOLOGY: ITS PLACE IN GENERAL EDUCATION.*

THE publication, in rapid succession, of the popular text-books on Physiology, the titles of which appear at the foot of this page, will be regarded with great satisfaction by all who take an interest in the promotion of sanitary science. The appearance of these works denotes, that the importance of a knowledge of the means of preserving health is gaining general—not, too hastily, to say universal—recognition; for there can scarcely be a stronger proof of the value attached to any branch of knowledge, than that a systematic attempt should be made to place its principles within the reach of all classes, and to introduce them into the instruction of youth.

As sanitarians, we must argue from the practical utility of the popular study of physiology. But there is another argument urged in favour of its pursuit—an argument which we fully appreciate, and which Dr. Lardner enforces in his peculiarly striking language.

"Of all the stores of knowledge", says this author, "there are surely none which should more pique our natural curiosity than those which open to us a view of animated nature. In these we have, so to speak, a selfish interest. So far as relates to our material being, we are ourselves one of the order of animals, among which we hold the highest place. Our organisation includes the most exalted specimen of Nature's work exhibited to us here below.

---

* Animal Physics; or the Body and its Functions familiarly explained. By DIONYSIUS LARDNER, D.C.L., formerly Professor of Natural Physiology and Astronomy in University College, London. London: Walton and Maberly. 1857.

Animal Physiology for Schools. By DIONYSIUS LARDNER, D.C.L. With 190 Illustrations. London: Walton and Maberly. 1858.

Atlas of Human Anatomy and Physiology. By WILLIAM TURNER, M.R.C.S. Eng., Demonstrator of Anatomy in the University of Edinburgh. Selected and arranged under the superintendence of John Goodsir, F.R.C.S., L. and E. Engraved and printed in colours by W. and A. K. Johnston. Edinburgh: W. and A. K. Johnston. 1857.

Handbook to Atlas of Human Anatomy and Physiology. By W. TURNER, M.R.C.S.Eng. Edinburgh: W. and A. K. Johnston. 1857.

Man; his Structure and Physiology; popularly explained and demonstrated. By R. KNOX, M.D., F.R.S.E., Lecturer on Anatomy, etc. With eight movable dissected Coloured Plates, and five Woodcuts. London: H. Baillière. 1857.

A Catechism of the Physiology and Philosophy of Body, Sense, and Mind; for use in Schools and Colleges, and in Private Study. By T. WHARTON JONES, F.R.S. London: Churchill. 1858.

Note on Teaching Physiology in the Higher Schools. By HENRY W. ACLAND, M.D., F.R.S., Regius Professor of Medicine in the University of Oxford. Pamphlet. J. H. and J. Parker. 1858.

The Museum of Science and Art. Edited by D. LARDNER, D.C.L. Articles —Air, Fire, the Eye, Spectacles, Man, Instinct, and Intelligence.

In no department of science has so extraordinary a body of knowledge been evolved. In none have results been obtained manifesting in a more striking manner the attributes of the Great Maker of All, than in the provisions made for the maintenance and continuance of the species which inhabit the earth, and for the moral and physical supremacy of man over them. . . . .

"The influence which the study of natural science exercises upon the intellectual faculties merits serious attention. The course of investigation through which the mind is conducted in such studies habituates it to ascend from effects to causes, yet never advancing a step without submitting the deductions of reason to the severe tests of experiment and observation. While such studies lead, therefore, to a habit of lofty speculation, they never permit the imagination to wander, inasmuch as the material verification is rigorously placed in juxtaposition with the speculative hypothesis. This, in a word, like every other branch of natural history prosecuted on just principles, exercises the mind better than any other in those methods of reasoning, without which all investigation is laborious and all exposition obscure." (*Animal Physics*, pp. 1-3.)

To insist on the importance of physiology as a branch of general learning, apart from its sanitary utility, may appear new and strange to many. Yet it is not new. The ancient philosophers—the acquirement of whose language has been made to take the foremost rank in our educational establishments, while their example in teaching practically useful subjects has been nearly utterly neglected—made this study a part of the instruction which they offered to the people. The *Timæus* of Plato is a prominent example of this; in it the structure and functions of the human body are set forth, according to the then prevailing ideas.

But the teaching of any branch of natural science cannot, in the nature of things, be limited in its effects to the mere development of the faculties of the mind. It must have a practical influence on the actions and habits of men :—that is to say (taking physiology as the example), the mere knowledge of the structure and functions of the body, and of the agencies which affect them, must lead all, who possess ordinary reason, to cultivate those influences which preserve health, and to avoid or remove those which injure it. Is it too much to ascribe in great measure to the influence of the popular teaching of natural science by the philosophers, the elaborate care which the ancient Greeks and Romans bestowed on the public health, as manifested in their numerous and excellent sanitary regulations ?

The incidental and indirect benefit, then, of physiology as a mere philosophical study would, we believe, be very great.

But our special duty is to point out the good, in regard to sanitary matters, which would arise directly from the diffusion of this branch of knowledge.

Prominent among the advantages that would arise, is the death-blow which would be struck at that power of imposture over ignorance, by which health and life are so ruthlessly sacrificed. Dr. Acland—whom we hail as a zealous and enlightened fellow-labourer—has a few very true words on this subject.

"I look to the increase of a general knowledge of physiology (and of hygiene which it implies) as one of the greatest benefits which will accrue through science to the temporal interests of mankind. Every form of quackery and imposture in medicine will in this way, and in this way only, be discouraged. It is, in great part, on this ground—on the ground of the future benefit to the people through the dissemination of a true perception of the *groundwork* of practical medicine, that I have laboured for many years to promote physiological knowledge in this university, among students holding whatever rank, and destined for whatever occupation." (*Note on Teaching Physiology*, p. 5.)

It is on ignorance, on the want of means of distinguishing truth from falsehood, or certainly from speculation, that the success of charlatans is supported. To a populace possessing a mere knowledge of the existence of the component parts of the human frame, and of their functions, but with little or no idea how these act on each other, or how they are affected by external influences, the extravagances of the quack sound as gospel; and he profits accordingly at the expense of his victims. The acceptance of absurd doctrines in physiology or in any other science is not, we think, due so much to a desire of receiving the doctrines themselves, as of receiving *something* which shall satisfy the natural craving of the mind for knowledge—true or false. Then, in the name of all honesty and common sense, let men's minds be filled with a knowledge of what is true. Let them be taught in simple language what is known, and practically useful, regarding circulation, respiration, digestion, the physiological processes of the body generally. Let them be taught that enough is known to enable men to preserve health, if care be taken not to impede Nature by officious interference. Let this instruction be firmly implanted, and there will be built up a fortification against the attacks of quackery, far stronger and more durable than any which could be raised by prohibitory legislation.

It is not merely as a preventive of charlatanic trifling with disease that a general knowledge of physiology is likely to be useful. The usages and fashions of our ordinary life are too often adopted without reference to natural laws; and consequently these laws are broken at least as often as they are observed. But the liability to this contradiction of Nature would be greatly diminished, probably altogether removed in time, by a general knowledge of what is incompatible with health and life. If, for instance, the necessity to respiration of allowing a free play of the thoracic apparatus were generally recognised, we should in a little time cease to hear of the mischiefs produced by tightly laced corsets; if the absolute need of a constant supply of pure air for the performance of the same function were known, it would no longer be a matter of popular indifference whether the poisonous gases of the carefully closed and perhaps overcrowded room, or the pure atmosphere, were inspired; if the doctrine of the alternation of repose and activity of organs were understood, less would be heard of overstrained muscle or overworked brain. These examples are merely suggestive, far from exhaustive.

The instances we have given of the benefits which would arise from popular instruction in physiology are general. But there are many special professions, other than the medical, in which this knowledge would find its useful application.

The sanitary condition of the *army* has already been made the subject of special comment in this number of the SANITARY REVIEW; and therefore it is not necessary to say much on the subject in this place. But to what extent the rate of mortality evidently traceable to *preventible causes* might be reduced, were the soldier instructed in the rudiments of physiology, we leave to the judgment of the well informed reader.

The *clergy* have the reputation of being among the most zealous and influential patrons of quackery. The influence which they possess arises in a great degree from the reputation of learning which is attached to them, as a result of their collegiate education. The people suppose their knowledge and judgment to be of universal application; and hence they are accepted as guides in matters on which they have as little correct information as those whom they mislead. The error lies in the system of education which has been generally followed in our universities and schools—a system far behind the requirements of the time. There is, however, a dawn of improvement in this direction, as we shall presently more plainly point out. The study of physiology would be important to the clergy if it only restrained them from doing mischief—if its influence on their actions were merely negative. But this knowledge would also be productive of positive good; for it would enable them to point out where infringements of natural laws were being committed, and how they might be obviated.

Members of the *legal* profession, especially in the higher grades, are often called on to decide questions involving a knowledge of the phenomena of life. It is true, that they ordinarily call medical evidence to their aid; but of the real value of this, they have little or no idea; and, if it be conflicting, they are liable to be utterly confounded. It cannot, then, be a matter for wonder, that they should sometimes adopt decisions entirely at variance with physiological facts. We say here little of those most abstruse phenomena, the mental functions; although these may, for all practical purposes, be included in our remarks. But, in regard to more obvious and tangible facts, how can a judge decide rightly, in cases where questions involving the several points arise, who does not know, for instance, the phenomena of the absorption of a poison, the circulation of the blood, the nature of the air which we breathe, or the mode in which the process of respiration is carried on? His decision, if correct, must be a piece of good luck; or it must be the result of the laborious exercise of common sense, operating in the midst of confusion, where a little instruction in common physiological principles would have made all clear.

To the conductors of *schools* a knowledge of physiology would be useful, in preventing them from committing many of the absurdities which are practised. In these institutions, especially, we believe, in girls' schools, there is too often a gross neglect of rational principles. As a rule, there is too much restraint of the natural lively impulses of children, while a rigid discipline of "Papa, potatoes, poultry, prunes, and prism", is enforced. This system is absurd and wrong enough in itself; but there are some institutions where the defiance of Nature is carried to a far greater extent. For instance, the American medical periodicals some months ago noticed an instance where, of the twenty-four hours, twelve were devoted to study, seven to sleep, three to meals, and two to exercise. And many are doubtless familiar with instances no less flagrant, in this land, and even in this metropolis of civilisation, where Nature is degraded to the position of a snubbed, ill fed, ill warmed, ill clad maid-of-all-work, in humble attendance, at respectful distance, on gentility. We may be supposed to refer to extreme instances; but we believe that, in general, children in schools are treated rather as machines to be charged with knowledge, than as members of the animal kingdom, living under the same physiological and physical laws as the young of any other species of the organised creation.

The possession of physiological knowledge would be useful to all classes of *manufacturers* and persons engaged in trade. It would give them some guidance as to the means of preventing or diminishing many of the diseases which commonly attend certain occupations, and which raise to a high rate the average mortality among the persons employed. It would teach how the labour must be proportioned to the capacity of the body for its endurance. "National health is national wealth", both generally and particularly; and a healthy body of workmen is obviously far more valuable than a set of semi-animate overworked imbeciles.

Many other examples of the practical application of a popular knowledge of physiology might be given: but the following quotation from Dr. Acland must suffice.

"General physiological questions will, in a few years, become so universally understood, that much ordinary literature will be unintelligible to those wholly unacquainted with them. Advanced physiological problems are already discussed in reviews, in this and other countries. Sanitary inquiries of all kinds come now within the range of town-councils, vestries, guardians, police, registrars, and officials in every class of society. *Cæteris paribus*, therefore, one versed in such subjects or intelligently grounded in them, is better prepared for office than one who is not." (*Note*, &c., pp. 4-5.)

When and how should the instruction in physiology which is here advocated be given? Obviously the best time for imparting an amount of information, at least sufficient for all ordinary purposes, is in youth. Physiology ought to be introduced as a branch of education into all our colleges and schools, public and private, for the female as well as for the male sex; and if the movement which has begun to be made in this direction receives the support it deserves, the outlines of the science of life will in a few years become as familiar in an educational establishment as the elements of history or geometry. Already we see pleasing signs of this recognition of useful knowledge. For some years past, indeed, there have not been wanting men—among whom the brothers Combe of Edinburgh hold an honourable place—who have laboured to disseminate a general knowledge of physiology. The Society of Arts, also, have endeavoured to promote the study of this science among the members of mechanics' institutes, by enrolling it among the subjects on which they hold annual examinations. The University of London has, from the beginning, required a knowledge of physiology from candidates for degrees in Arts. In the University of Oxford, we learn from Dr. Acland's pamphlet, dated December 1857, that

"In the Grammar School at Magdalen College, the head master, the Rev. J. E. Millard, has permitted the introduction of physiology during the past term as an experiment. An examination was held at the end of term by the teacher, Mr. Griffiths, of Jesus College."

This example is highly creditable to the University of Oxford; and we trust to see it followed everywhere; for it is from the systematic adoption of physiology as a branch of general education that useful results must be expected, rather than from the desultory manner in which this knowledge has hitherto been imparted to the public. Our large public schools will bear much reform in this direction. Even in the Royal Medical Benevolent College, a primary object of which is the education of sons of medical men, and which is chiefly managed by members of the profession, physiology (and, indeed, all natural science) is strangely omitted from the regular curriculum of study, and is apparently as completely ignored as at Harrow or Eton. These great institutions would gain a vast addition to their present reputation by the spontaneous introduction of natural science; that they *must* sooner or later introduce it, will be an inevitable result of the educational movement now in progress.

There are no doubt many objections which may be raised against the introduction of physiology into schools; but none which may not be met. It may be said, that it would be adding to the already overloaded curriculum of education; that it would be in itself an infringement of the physiological principle that the mind (like any other organ) cannot be overburdened without danger. All this might be quite true, if it were proposed merely to superadd physiology to the mass of education at present imparted—if the hours required for healthy corporeal exercise were to be abridged. But what is demanded, is that physiology should occupy a fair share of the time now devoted to the acquisition of knowledge less practically useful, and not at all superior as means of mental discipline.

Again, it may be argued that a knowledge of physiology is necessary only to the physician—to him whose special duty it is to aid nature in repairing the injuries suffered by the body. Without doubt, to the man of physic is requisite the most intimate knowledge which can be attained of the structure and functions of that body, on which, when it is diseased, he employs the resources of his art: but this by no means precludes that every man should know so much of his own mechanism as is necessary for maintaining himself in health. To the physician belongs especially the task of attending to the repair of a deranged mechanism: to every possessor of that mechanism belongs the duty of preserving it, as he best can, in working order.

Popular instruction in physiology should aim at imparting a correct knowledge of leading facts and principles rather than of details. Dr. Lardner well says:

"It is not necessary to a liberal education to be able to pronounce upon the nice distinctions which separate species from species, nor to trace the course of each artery and nerve which traverses the organs of the body. Such details would encumber the memory of the general student, without leaving upon it any durable or useful traces. The knowledge which is desired is of another kind. The general structure of the human body and its organs; the principal functions by which they are sustained and nourished; the most striking relations between them and the corresponding organs of inferior species: the manner in which the functions are exercised; and the relations between the varying structure of the organs and the habitudes of life of the species to which they belong, constitute knowledge which, once well and soundly acquired, can never be forgotten, and which, while it suffices for those whose pursuits in life do not necessarily connect them with the prosecution of the natural sciences, serves as the base of the special studies of those who engage in professions, with which these sciences are inseparably connected." (*Animal Physics*, p. 2.)

Before concluding, a word remains to be said on the several works which have been enumerated at the beginning of this article.

Of Dr. Lardner's *Animal Physics* we have in a former number spoken in terms of commendation, as a first-class popular treatise on physiology.

The *Animal Physiology for Schools* is evidently an abridgement of the *Animal Physics,* reduced to a size which places the instruction contained in it within the reach of youth, for whom the larger work, excellent as it is for adults, would be too elaborate. The smaller work combines the qualities of accuracy and clearness of description with cheapness in price. The author expresses a hope

"That this volume may be the means of extending instruction in the first notions of Animal Physiology into ladies' schools. . . . In the selection of subjects and in the mode of treating them, the author has kept this constantly in view, introducing nothing which may not with perfect propriety and advantage be brought before the youngest female minds."

We must not omit to say that the book is illustrated with one hundred and ninety wood engravings: and that at the end of the volume is added a glossary, containing brief explanations of the less familiar terms. We expect that, as the importance of physiology becomes appreciated by the public, this little work will meet with great and merited favour.

Mr. Turner's *Atlas* and *Handbook of Human Anatomy and Physiology* is also a work of much merit, but far too expensive to be likely to come into very general use in schools. Its principal value will be to the teacher, who may, with much profit, use the *Atlas* for the purpose of giving occasional anatomical demonstrations to his pupils.

Dr. Knox's treatise is, like that of Mr. Turner, confined to human anatomy and physiology. It is an elegant book, chiefly remarkable for the manner in which it is illustrated.

"The *Atlas* contains eight plates or engravings representing, as in a medallion or coin, both sides; that is, the direct and the reverse; thus giving to the engraving, in so far as the art can give, the effect of a natural representation. But it also contains other engravings, in which in addition, certain figures have been cut into or dissected, and superimposed, in order to preserve to the eye different layers and different surfaces of the same organ. . . . The layers or laminæ, movable and articulating upon the principal figure, may be raised like the leaves of a book: and the reader may, with their assistance, study the apparatus or assemblage of organs contributing to a function, dissected layer by layer."

Mr. Wharton Jones's intention has been good; but we cannot speak with very high praise of his performance. Indeed, we feel that, as an acknowledged physiologist of excellent repute, he has not on this occasion sufficiently guarded his well earned fame. His *Catechism* is totally deficient in that indispensable adjunct to a popular work on physiology—representations of the things described; for anatomy and physiology cannot be usefully taught by words alone. For our own part, moreover, we object altogether to the system of instruction by catechisms, as one fitted for implanting in the mind of the learner words rather than knowledge, and set phrases rather than ideas.

